# "Reductio Ad Absurdum"

**Published:** 1909-11-27

**Authors:** Frederick Dixon

**Affiliations:** 23 and 24 Clun House, Strand, W.C.


*To the Editor of* The Hospital.

### "REDUCTIO AD ABSURDUM."

*To the Editor of* The Hospital.

Sir,—The article in your issue of the 6th inst., entitled "Reductio ad absurdum," deals, in its criticism of Christian Science, with so many points that it would be impossible to cover them all in the space of a single letter. Will you permit me, however, to point out that if you are allowed to state the premises of those from whom you disagree, in your own way, it is not particularly difficult to reduce them to an absurdity? It has been done with triumphant effect in our own time by the rationalistic critics of Christianity, just as it was done, a couple of centuries or so ago, by the coxcombs who, as Huxley said, vanquished Berkeley with a grin. It is, however, never effective unless the premises themselves are ridiculous, and in this case they can be trusted to destroy themselves, instead of attracting adherents in an ever-increasing progressive ratio, as has been the case with Christian Science during the last forty years.

However this may be, I imagine the main contention of the article to be that Christian Science workers attempt to heal the sick without making any diagnosis. Now will you allow me to say that this is quite incorrect? Christian Science workers make a very searching diagnosis; but they make it, naturally, in a very different way to an ordinary doctor. It is the teaching of Christian Science that all sickness is the result of a mental cause. You may call this nonsense if you choose; but it is, so far as it goes, the teaching of all the great advocates of idealism in natural science. If this premise is correct, it follows that to heal any disease the mental cause must be destroyed, and it is to the destruction of the mental cause that the Christian Scientist directs his efforts. The diagnosis of the physical phenomena, which he maintains are but the subjective condition of the human mind, does not consequently, interest him. They constitute nothing but the effects of belief in certain physical laws which it is his business to prove inoperative, and consequently not law. It is simple enough to dismiss his premises as ridiculous, but it cannot possibly seem more grotesque to the medical profession than the theory that the sun was stationary did to the mediæval ecclesiastics.

But, replies the doctor, Christian Science does not heal. The simple fact is that the Christian Science movement has been built up on healing. One thing you cannot fool the sick about, and that is as to whether they get well or not. The man in the Bible who confounded the Pharisee with the *ne plus ultra,* "One thing I know, that, whereas I was blind, now I see," is typical of humanity. For upwards of forty years Christian Science has been proving its faith by its works, and the result is seen in the power of the movement to-day. The Christian Scientist does not, of course, succeed in always healing his patient, but neither does the ordinary medical practitioner. To attempt to judge a system by the failures of its exponents is about the most futile thing in the world. To accept the statement of a doctor, in any definite case, that medical science would have succeeded where a Christian Scientist has failed, is not only to make one of two parties to a dispute the judge of the issue, it is to maintain something which, in similar circumstances, is being disproved every day. Yours truly,

Frederick Dixon.

23 and 24 Clun House, Strand, W.C.
November 8, 1909.

